# Dentinogenic Ghost Cell Tumor in an Elderly Female: A Rare Case Report

**DOI:** 10.1155/crid/5758655

**Published:** 2025-12-07

**Authors:** Akunchan Shrestha, Iccha Kumar Maharjan, Pragya Regmee, Abhinaya Luitel, Mehul Rajesh Jaisani, Neetu Jain, Shristi Maharjan, Asha Rai, Regina Dahait, Numa Limbu

**Affiliations:** ^1^ Department of Oral Medicine and Radiology, College of Dental Surgery, B.P. Koirala Institute of Health Sciences, Dharan, Nepal, bpkihs.edu; ^2^ Department of Oral and Maxillofacial Surgery, College of Dental Surgery, B.P. Koirala Institute of Health Sciences, Dharan, Nepal, bpkihs.edu; ^3^ Department of Oral Pathology, College of Dental Surgery, B.P. Koirala Institute of Health Sciences, Dharan, Nepal, bpkihs.edu; ^4^ B.P. Koirala Institute of Health Sciences, Dharan, Nepal, bpkihs.edu

**Keywords:** dentinogenic ghost cell tumor, dentinoid material, ghost cells

## Abstract

Dentinogenic ghost cell tumor (DGCT) is an uncommon odontogenic neoplasm that accounts for less than 0.5% of all tumors with odontogenic origin. Its rarity, along with identical radiological and clinical features as other odontogenic lesions, often makes the diagnosis challenging. The current case report discusses the clinical, radiological, and histopathological features of DGCT in a 71‐year‐old female patient presenting with facial swelling and pain in the maxillary anterior region. Clinical examination revealed a hard, tender, localized swelling from the infraorbital to the zygomatic regions. Intraoral exam revealed a sessile swelling buccopalatally extending and involving the labial vestibule and hard palate. Imaging showed a unilocular, expansile lytic lesion with hyperdense contents and a thinned cortex, suggestive of odontogenic pathology. Histopathology confirmed the diagnosis of DGCT on the basis of ghost cells with calcification, ameloblastoma‐like epithelial islands, and dentinoid material in connective tissue. Treatment was done by wide local excision with a maxillary obturator prosthesis. Six‐month postoperative follow‐up showed no recurrence or complication. This case highlights the importance of combining clinical, radiological, and histopathological examination to diagnose rare odontogenic tumors like DGCT. Despite being benign, the tumor′s aggressive potential demands radical resection and extended follow‐up for monitoring recurrence. The case also highlights the importance of raising awareness among clinicians to achieve optimum diagnostic yield and patient outcomes.

## 1. Introduction

Odontogenic cysts and tumors exhibit great diversity in their histological characteristics and their behavior patterns. Complete clinical, radiographic, and histopathological examination is necessary in order to establish an accurate diagnosis. Gorlin et al. described the calcifying odontogenic cyst (COC) as a specific pathological entity for the first time in 1962 [1]. The entity was presented as a well‐circumscribed cystic or solid mass of odontogenic origin with follicular ameloblastoma features along with ghost cells. The lesion histologically resembled the cutaneous calcifying epithelioma of Malherbe, also referred to as “pilomatricoma,” and was thought to be its oral equivalent. The lesion was categorized in 1971 by the World Health Organization (WHO) as a nonneoplastic cystic lesion and was described as a “nonneoplastic cystic lesion in which the epithelial lining has a well‐defined basal layer of columnar cells, a second layer that is often several cells thick and stellate reticulum‐like, and masses of ghost epithelial cells that may be found within the epithelial cyst lining or the fibrous capsule.”

Dysplastic dentin can potentially be deposited beside the basal cell layer of the epithelium [2]. Prætorius et al. [3] maintained that COCs are composed of two different entities: a cyst and a neoplasm. Later, in 2004, the WHO, following this monistic view, classified all COCs as neoplasms, naming them “calcifying cystic odontogenic tumors” (CCOT), which has resulted in uncertainty about their classification as cysts or tumors. Later, the cystic lesion was named COC, and the neoplastic variant was designated as “dentinogenic ghost cell tumor” (DGCT) [4]. It is an extremely rare benign odontogenic tumor, which commonly shows solid proliferation in a central/intraosseous or peripheral/extraosseous location [5, 6]. DGCTs consist of epithelial neoplastic islands that resemble ameloblastoma with ghost cells and dentin [6, 7] As per the 2017 WHO classification of odontogenic tumors and cysts, DGCT falls under benign tumors of mixed (epithelial–mesenchymal) origin [8]. This has not been altered in the WHO′s 2022 update [9].

To date, there has been one case report of DGCT reported from Nepal, which reflects the rarity of the disease.

## 2. Case Report

A 71‐year‐old female patient visited the Department of Oral Medicine and Radiology of BPKIHS, Dharan, with a complaint of facial swelling localized to the right upper frontal area that had persisted for 10 days. The onset of swelling was acute, and it continued to increase in size over time.

**Figure 1:**
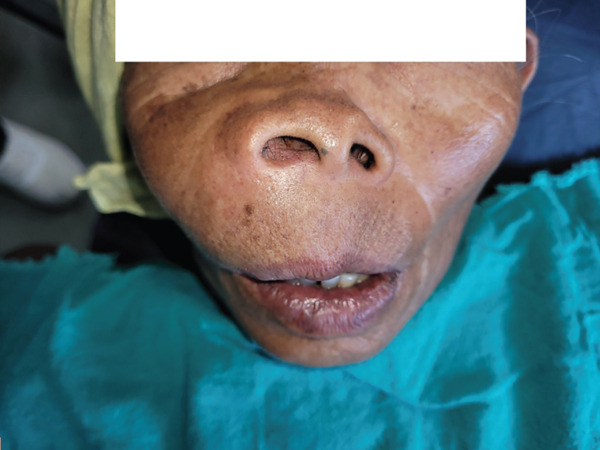
Extraoral view of the patient showing swelling in the midface region, localized to the right upper anterior area.

The associated pain was acute in duration, constant, progressive, and throbbing in character, mild in intensity (numerical rating scale: 3/10), and nonradiating, without any relieving or aggravating factors. No associated symptoms, such as trauma, functional disturbances (difficulty in speech, difficulty in chewing, or difficulty in swallowing), blurred vision, nasal epistaxis, significant weight loss, recurrence, or remission of swelling, were present. Her medical, dental, family, and personal history were noncontributory. Facial asymmetry was observed on extraoral examination, with a single, unilateral, ill‐defined swelling in the infraorbital and zygomatic areas. The swelling had an anteroposterior dimension from the philtrum to the lateral aspect of the zygomatic bone and a superoinferior dimension extending 0.5 cm below the medial canthus of the eye, going on to the upper lip and to the right corner of the mouth. It was accompanied by right nostril flaring, nasolabial fold obliteration, and fullness of the upper lip. Swelling was oval, approximately 6 × 5 cm^2^, firm, tender upon palpation, and nonpulsatile, with normal overlying skin and margins (Figure 1). There was no loss of sensation over the swelling and no lymph node enlargement.

Intraoral examination revealed a solitary, localized, well‐defined, smooth, sessile swelling on the right side that extended buccopalatally from the labial vestibule to the hard palate. In the anteroposterior direction, it extended from the distal end of 22 to the distal end of 17 and in the superoinferior direction from the marginal gingiva to the depth of the vestibule.

**Figure 2:**
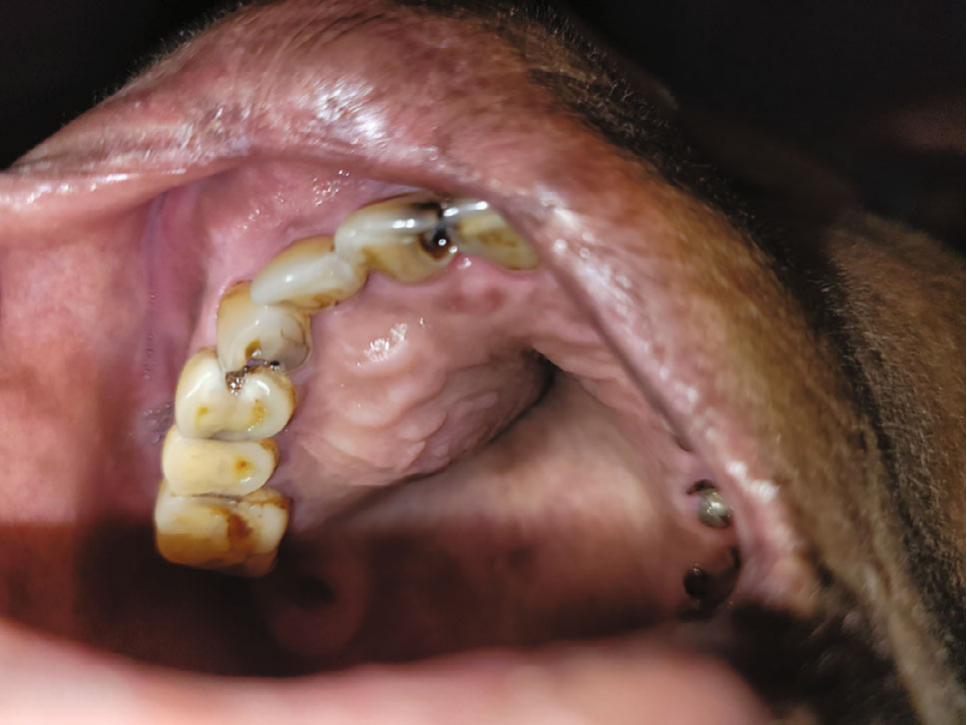
Intraoral view revealing swelling in the right maxillary region extending from labial vestibule to hard palate.

**Figure 3:**
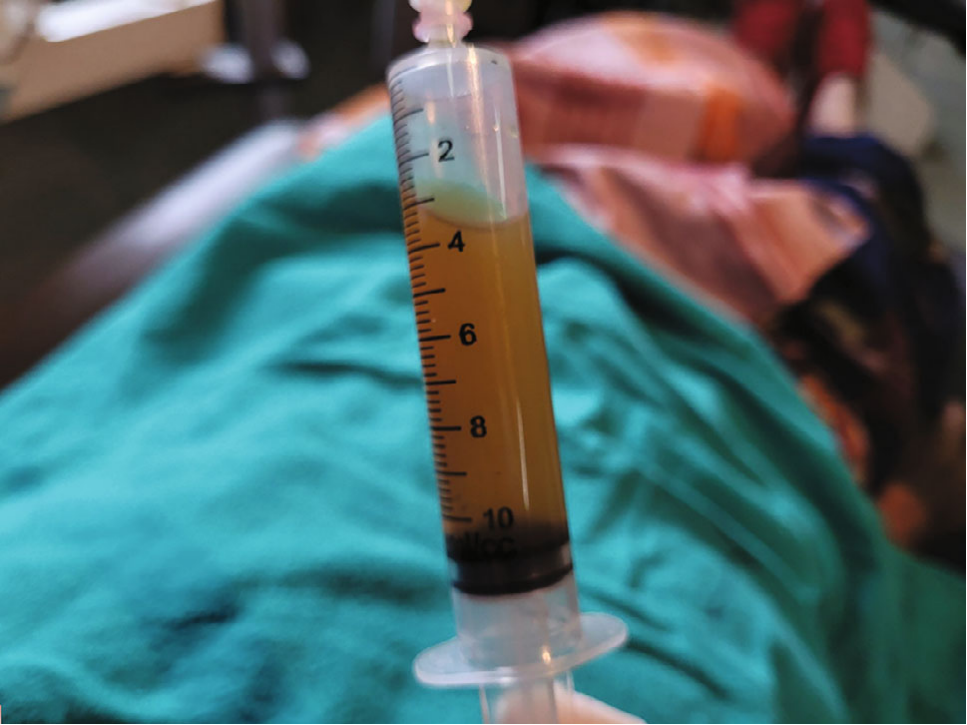
Aspirate obtained from the lesion, showing straw‐colored fluid.

**Figure 4:**
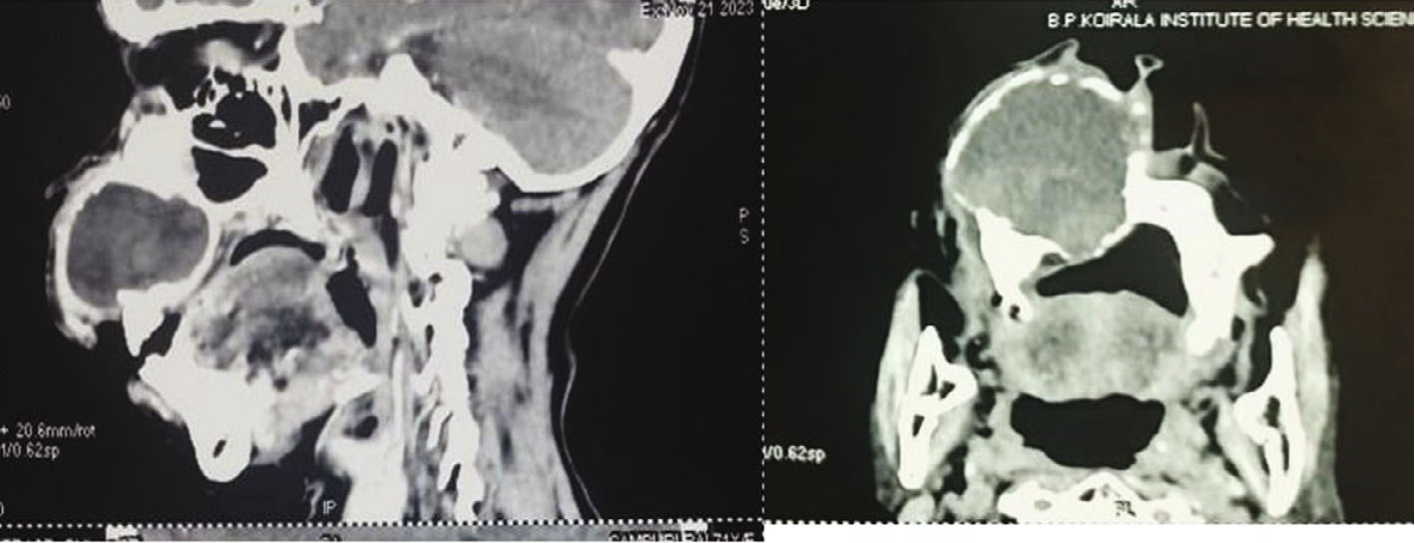
Contrast‐enhanced CT (CECT) scan (sagittal and axial sections) demonstrating an aggressive lesion with buccal alveolar expansion, cortical bone thinning, and multifocal erosion.

**Figure 5:**
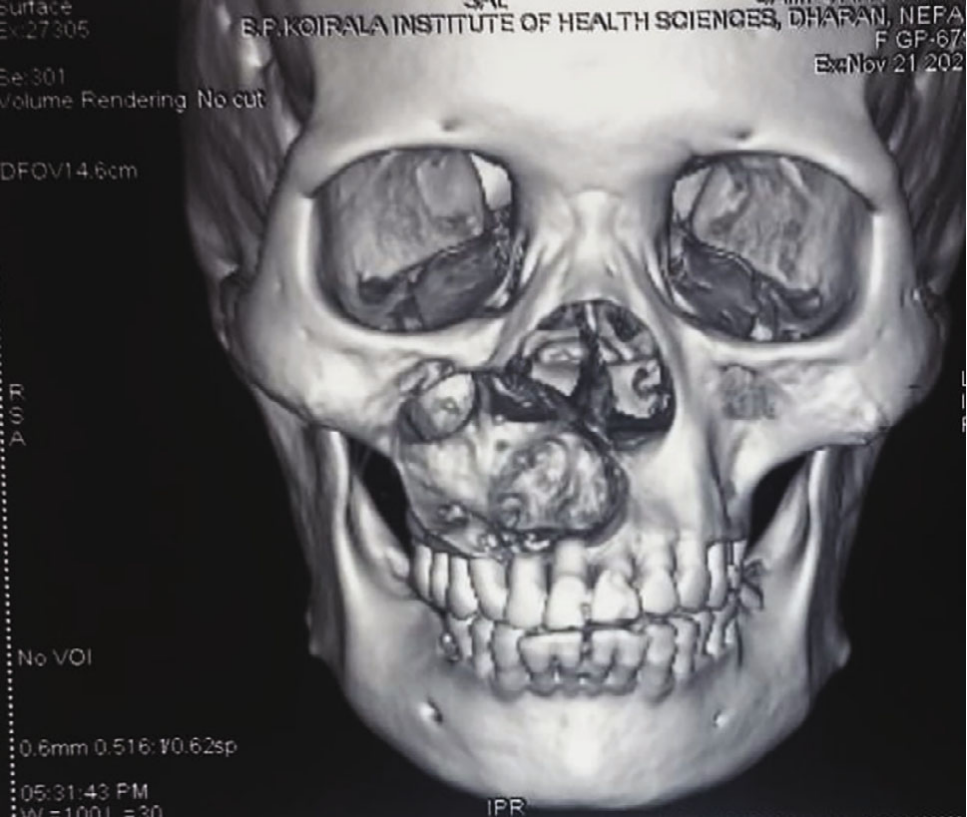
Three‐dimensional reconstruction of the lesion highlighting its extent and structural involvement.

The swelling measured 43 × 52 mm^2^ on the palatal and 29 × 42 mm^2^ on the labial aspect with a coarsely oval shape. The mucosal covering over it was intact, with no signs of secondary changes such as ulceration or discharge. Upon palpation, the swelling was soft to firm in consistency, tender, nonpulsatile, and nonfluctuant (Figure 2). There were no occlusal disturbances. Aspiration yielded straw‐colored fluid (Figure 3). Electric pulp testing confirmed the vitality of all the involved teeth (21, 11–16). A provisional diagnosis of right maxillary nasolabial cyst was made, with differential diagnoses of odontogenic keratocyst, adenomatoid odontogenic tumor, COC, and ameloblastic fibro‐odontoma. Contrast‐enhanced computed tomography (CECT) scan revealed a single, unilocular, expansile lytic lesion with nonenhancing hyperdense content in the right maxilla, involving the alveolar process of 11, 12, and 13. It had a well‐defined, partially corticated margin and predominantly hypodense internal structure. The buccal expansion of the alveolus and thinning with erosion were noted at multiple sites (Figures 4 and 5). The differential diagnoses observed in the radiographic examination were odontogenic keratocyst, adenomatoid odontogenic tumor, ameloblastic fibro‐odontoma, COC, and calcifying epithelial odontogenic tumor.

An incisional biopsy revealed islands of odontogenic epithelial cells that were embedded in a formed connective tissue stroma. There was a prominent occurrence of ameloblastoma‐like follicles with peripheral columnar, polarized basal cells featuring hyperchromatic nuclei. Ghost cells, which are eosinophilic cells without nuclei but with their basic cellular contour, were found within epithelial islands and also within connective tissue (Figures 6 and 7). Calcifications within ghost cells and dentinoid‐like material, along with cholesterol clefts and arcade‐like epithelium, were noted, leading to the diagnosis of DGCT. The surgical excision was performed, confirming the diagnosis again to be DGCT, following which a maxillary obturator was placed.

Figure 6H&E stain. (a) 10× and (b) 40× nest and islands of odontogenic epithelium resembling ameloblastoma along with ghost cells ranging from individual to large aggregated mass. Tissue resembling stellate reticulum is present at the center.

**Figure:**
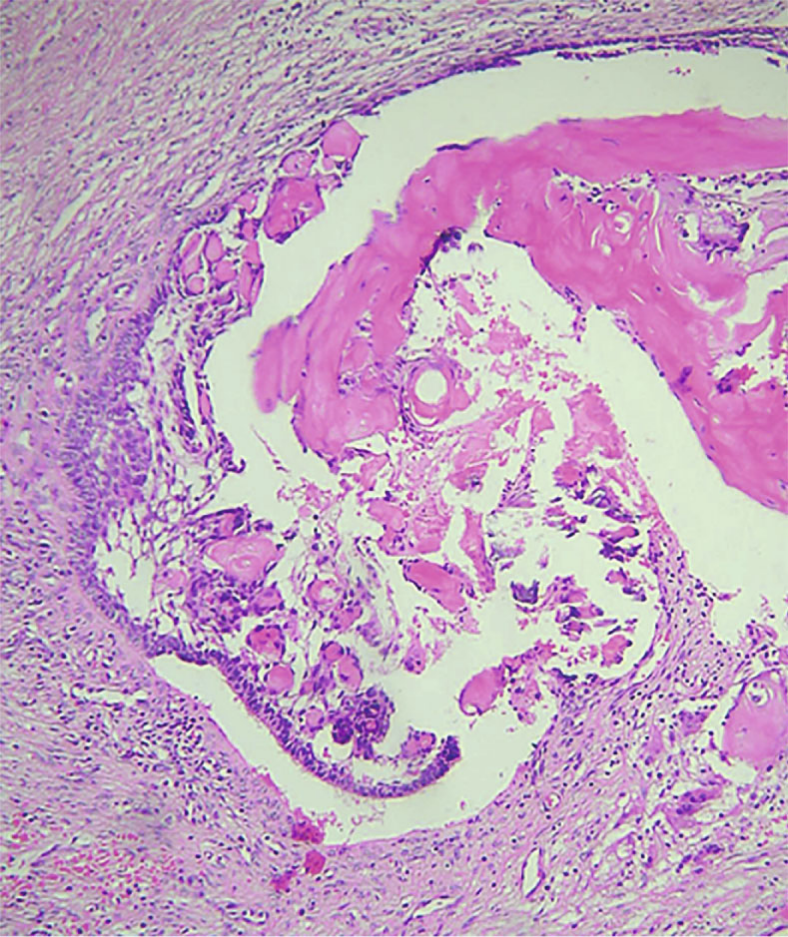
(a)

**Figure:**
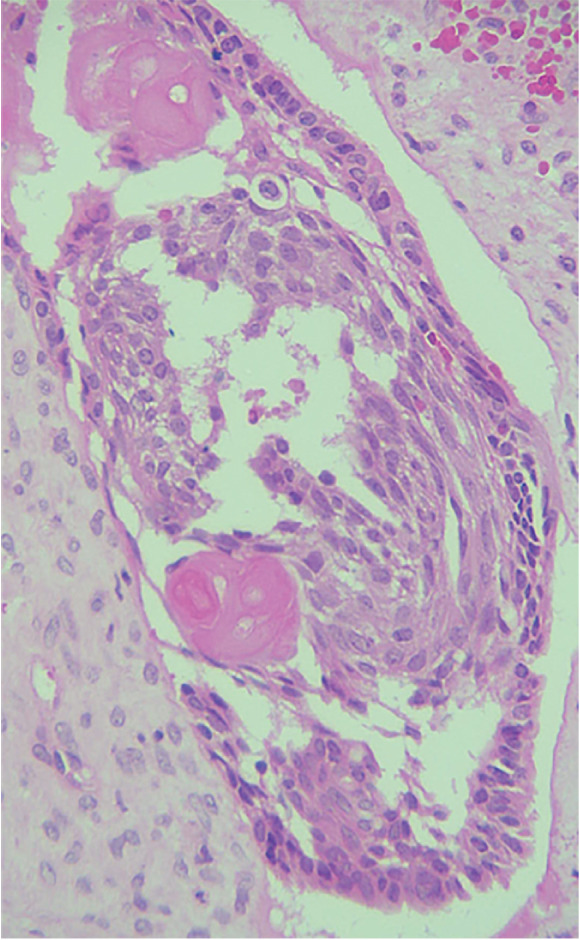
(b)

Figure 7H&E stain. (a) 10× areas of irregular eosinophilic masses suggestive of dentinoid deposit. (b) 10× mineralization of dentinoid within connective tissue.

**Figure:**
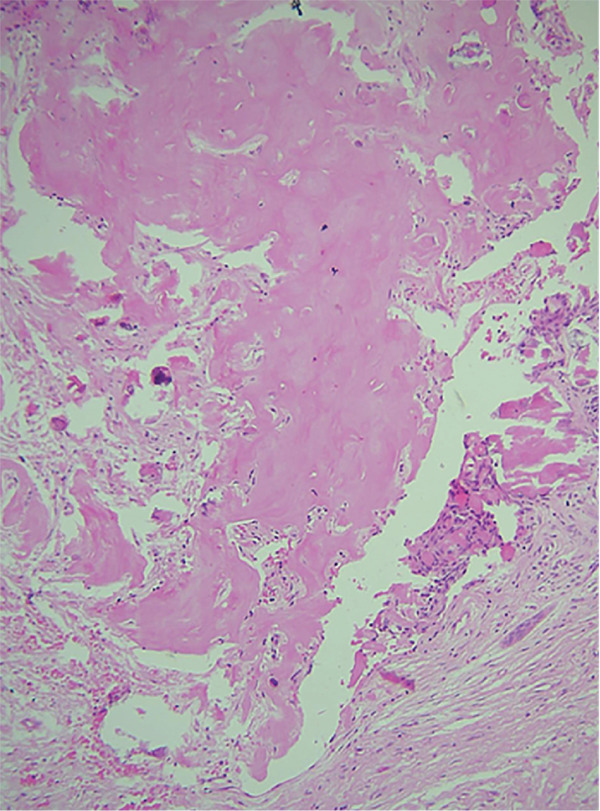
(a)

**Figure:**
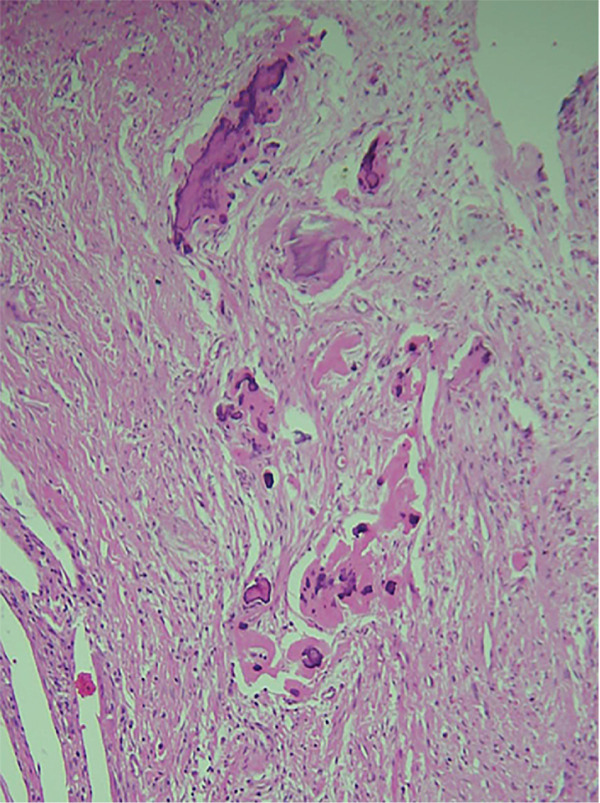
(b)

The patient was followed up for 6 months with no postoperative complications or signs of recurrence.

## 3. Discussion

In 2005, WHO classified DGCT as an odontogenic tumor. According to WHO 2017 and 2022, DGCT is defined as “a benign but locally infiltrating neoplasm of odontogenic epithelium.” It is uncommon, with less than 60 cases described in the literature, occurring mostly in Asian populations [10]. They are solid lesions with occasional malignant behavior. DGCTs are intraosseous in the majority of cases, that is, 83%, and 17% are peripheral lesions, predominantly in the gingiva [4].

Intraosseous DGCTs have been reported to occur in a vast age group of 8–80 years with slight male predilection, more common in the mandible than maxilla. They can recur even after resection as they are more aggressive, with a highly infiltrative growth pattern. However, the extraosseous types are less common, occur at the sixth decade of life, are common in the alveolar mucosa or gingiva, and exhibit limited growth potential. Both variants show similar histopathological features [11]. In our case, a 71‐year‐old woman presented with a lesion in the anterior maxilla, which does not match the classical demographic and clinical characteristics of this entity completely. The intraosseous DGCT presents with a diameter of 1 cm to over 10 cm. The clinical presentations are swelling, facial asymmetry as a result of jaw enlargement, and the occasional maxillary sinus obliteration or infiltration into adjacent soft tissues. The swelling may either be painful or painless and at times may be associated with pus discharge, tooth mobility, or displacement [12].

Radiographically, DGCT presents as unilocular/multilocular radiolucency with scattered radiopacities suggestive of calcifications. It has a bicortical expansile nature, while in some cases, there is a presence of impacted teeth, along with their root resorption, displacement, and root resorption of adjacent teeth. Some of the lesions show ill‐defined borders and maxillary lesions extending up to the orbital floor [13]. When there is no internal calcification and the lesion is pericoronally positioned, it may not be differentiable from a dentigerous cyst. They tend to mimic other odontogenic lesions, such as adenomatoid odontogenic tumors, ameloblastic fibro‐odontomas, and calcifying epithelial odontogenic tumors [14].

The differential diagnosis of DGCT usually includes COC, adenoid ameloblastoma, and ghost cell odontogenic carcinoma. DGCT is differentiated from COC based on the presence of solid characteristics, ghost cells, and dentinoid exceeding 1%–2%, as observed on histopathological evaluation [15]. Adenoid ameloblastoma shows cribriform and ductal arrangements of basal ameloblast‐like cells, with varying amounts of dentinoid and ghost cells. In contrast, the DGCT lacks cribriform and ductal arrangements [16]. Ghost cell odontogenic carcinoma displays features similar to those of DGCT but with cytologic features of malignancy, including cellular atypia and pleomorphism, conspicuous mitotic activity, necrosis, and infiltration into adjacent tissues [17]. Based on the above histopathological findings, a diagnosis of DGCT was made.

Furthermore, numerous immunohistochemical studies have been documented pertaining to this lesion′s profiling. Odontogenic epithelium is a significant component of DGCT which has the potential to express epithelial markers CK 5, CK 7, CK 8, CK 14, and CK 19. Dentinoid material also shows positivity for the p53 marker [18]. In our practice, due to a lack of facilities, these tests were not possible to conduct.

Intraosseous DGCTs have a higher potential for malignant transformation as compared to their extraosseous counterparts, which are less likely to progress to malignancy [13]. If the tumor is radiologically ill‐defined, aggressive local resection would be advocated. Tumor complete excision might be achieved with segmental resection or block excision, based on its anatomic spread or size. Therefore, treatment strategies for intraosseous DGCTs require more extensive surgical procedures, such as bone resection, while extraosseous DGCTs are usually treated with simpler excision of the soft tissue lesion.

Following conservative surgical management, a 73% recurrence rate has been reported following 1–20 years of follow‐up, whereas in those managed with radical surgical procedures, the recurrence rate drops to 33% [19]. Information on other forms of therapy beyond surgical management is still limited. As findings in this case contribute to the limited literature on this entity, limitations such as the single case nature, short follow‐up duration, and absence of immunohistochemical analysis should be acknowledged. Future studies with larger case series, longer follow‐up, and comprehensive histopathological and molecular evaluation are needed to better understand the biological behavior and optimize management strategies for DGCT.

## 4. Conclusion

DGCT, being the solid variant of COC, is an uncommon odontogenic neoplasm and represents 2%–14% of COCs. The case here is reporting a central DGCT in a 71‐year‐old female with a maxillary anterior lesion that was treated with wide local resection without recurrence to date. With its rarity and limited literature, increased clinical awareness by practitioners diagnosing and treating head and neck pathology is paramount in attaining the best patient outcomes.

## Ethics Statement

The authors have nothing to report.

## Consent

Written informed consent was obtained from the patient to publish this report in accordance with the journal′s patient consent policy.

## Conflicts of Interest

The authors declare no conflicts of interest.

## Funding

The authors have nothing to report.

## Data Availability

The data that support the findings of this study are available from the corresponding author upon reasonable request.
